# Is Takotsubo syndrome induced by patent ductus arteriosus occlusion?

**DOI:** 10.1186/s12872-024-03788-0

**Published:** 2024-03-02

**Authors:** Shuhao Li, Senyan Yang, Shujing Zhou, Shuang Zhang, Xueying Li, Haibo Zhang, Ping Ren, Yanwu Liu, Ying Liu, Yuanguo Chen

**Affiliations:** 1 Department of Cardiovascular Medicine, the Ya’an People’s Hospital, Sichuan Province, People’s Republic of China; 2 Faculty of Medicine, University of Debrecen, 4032 Debrecen, Hungary; 3 MianYang Central Hospital, Sichuan Province, People’s Republic of China; 4 Second People’s Hospital of Ya’an City, Sichuan Province, People’s Republic of China; 5 Department of Cardiology, Sixth Medical Center, PLA General Hospital, Beijing, People’s Republic of China

**Keywords:** Takotsubo syndrome, Congenital heart disease, Patent ductus arteriosus, Interventional occlusion; case report

## Abstract

Takotsubo syndrome (TTS), commonly referred to as "broken heart syndrome," is a distinctive form of acute and reversible heart failure that primarily affects young to middle-aged individuals, particularly women. While emotional or physical stressors often trigger TTS, rare cases have been linked to interventional procedures for congenital heart disease (CHD). Despite its recognition, the exact causes of TTS remain elusive. Research indicates that dysregulation in autonomic nerve function, involving sympathetic and parasympathetic activities, plays a pivotal role. Genetic factors, hormonal influences like estrogen, and inflammatory processes also contribute, unveiling potential gender-specific differences in its occurrence. Understanding these multifaceted aspects of TTS is crucial for refining clinical approaches and therapies. Continued research efforts will not only deepen our understanding of this syndrome but also pave the way for more targeted and effective diagnostic and treatment strategies. In this report, we conduct an in-depth analysis of a case involving a TTS patient, examining the illness progression and treatment procedures. The aim of this analysis is to enhance the understanding of TTS among primary care physicians. By delving into this case, we aspire to prevent misdiagnosis of typical TTS cases that patients may present, thereby ensuring a more accurate diagnosis and appropriate treatment.

**Supplementary Information:**

The online version contains supplementary material available at 10.1186/s12872-024-03788-0.

## Introduction

TTS is an acute and typically reversible syndrome of heart failure [1]. The majority of patients manifest with severe chest pain, ST-segment elevation on Electrocardiogram (ECG), and mild elevation of troponin levels. A minority of patients present solely with arrhythmia [1]. Notably, coronary angiography usually reveals no apparent coronary artery disease. Since its initial report in 1990, TTS has garnered increasing attention among medical professionals, resulting in a rise in TTS diagnoses. Current studies indicate that sympathetic overactivation appears to play a central role in its pathophysiological mechanism. Emotional and pain stimulation, along with other factors, may contribute to the development of this condition [2, 3]. Over years, a considerable number of physicians have had access to information about TTS through medical textbooks while their familiarity with this ailment remains inadequate in practice. A subset of medical practitioners may necessitate supplementary clinical exposure to attain a comprehensive comprehension of TTS. The author endeavors, through the meticulous analysis of this case, to enhance the depth of understanding among primary healthcare providers concerning TTS. This initiative is pivotal in averting misdiagnosis of patients presenting typical symptomatic profiles, thereby improving the overall clinical acumen within the medical community.

### Case summary

A 40-year-old female patient was admitted to our hospital to undergo interventional treatment for patent ductus arteriosus (PDA). Cardiac catheterization revealed the presence of pulmonary hypertension without any history of chronic illness. Following the occlusion procedure, the patient experienced symptoms such as dizziness, vomiting, bradycardia, and increased aortic pressure. Later on, she developed signs of left heart failure, including dyspnea. Transthoracic echocardiography (TTE) confirmed a left ventricular ejection fraction (LVEF) of 31% (not shown). Approximately 12 h post-occlusion, the patient suddenly experienced ventricular fibrillation, which was immediately resolved with electrical defibrillation. The ECG displayed progressive T-wave reversal and a significant prolongation of the QTc interval. Subsequent TTE, Coronary Angiography and Left Ventriculography led to the final diagnosis of TTS. After discharge, the patient was prescribed Angiotensin Converting Enzyme Inhibitor (ACEI) and beta blockers. At the six-week follow-up, the LVEF and ECG returned to normal.

### Learning point

PDA occlusion may serve as a trigger for TTS. Pain stimulation resulting from PDA occlusion and dysregulation of autonomic nerve function may contribute to its pathogenesis. Arrhythmia was the primary manifestation of TTS in this case, suggesting that TTS could be a significant contributor to sudden death [1].

### Timeline

#### Thirty days prior to admission

TTE confirmed the diagnosis of PDA. The diameter of the aortic side of the ductus arteriosus was approximately 12 mm, and the diameter of the pulmonary side was around 8 mm. The LVEF before PDA occlusion was 51%.

#### One day prior to the procedure

Physical examination revealed a continuous cardiac murmur graded 4/6 at the left sternal margin. Biomarkers, ECG and TTE were normal.

### Occlusion procedure

Local infiltration anesthesia was administered using 5% Lidocaine. The procedure commenced with the puncturing of the right femoral artery and vein, facilitating comprehensive cardiac catheterization. Subsequent measurements included recording pressures in the pulmonary artery, right ventricle, and aorta (Table 1), as well as oxygen saturations of aortic, mixed venous, and pulmonary blood (Table 2). Additionally, the pulmonary to systemic blood flow ratio (Qp/QS = 1.55) was meticulously calculated to assess hemodynamic parameters. Then, aortic angiography was performed using a 6F Pigtail catheter (Cordis, USA). This step was crucial for measuring the diameters of the ductus arteriosus at both the aortic side (12 mm) and pulmonary side (8 mm) (Video 1). After angiography, a 10F delivery catheter (Shenzhen Xianjian, China) was employed for the occlusion of the PDA. The catheter navigated through the pulmonary artery into the PDA and then into the descending aorta (Video 2). An 18 mm occlusion device (Shenzhen Xianjian, China) was deployed at the ductus arteriosus (Video 3). Its stability was affirmed through a pull-test, and TTE showed no residual left-to-right shunt, indicating an ideal positioning and morphology of the device. The disappearance of the murmur, as confirmed through auscultation, marked the successful completion of the procedure.

**Table 1:** The pressures (in mmHg) measured during cardiac catheterization in the cardiac chambers, pulmonary artery, and aorta

Variables	Pre-Occlusion	Post-Occlusion
Pulmonary artery pressure(mmHg)	62/26(37)	42/10(20)
Right ventricular pressure(mmHg)	65/21(39)	45/3(18)
Aortic pressure(mmHg)	160/75(103)	200/93(128)

**Table 2:** Oxygen saturations measured during cardiac catheterization in the cardiac chambers, pulmonary artery, and aorta

Variables	Blood oxygen saturation
Aorta artery(%)	97
Mixed venous(%)	72
Pulmonary artery(%)	81

### Five minutes after the procedure

**Fig. 1:**
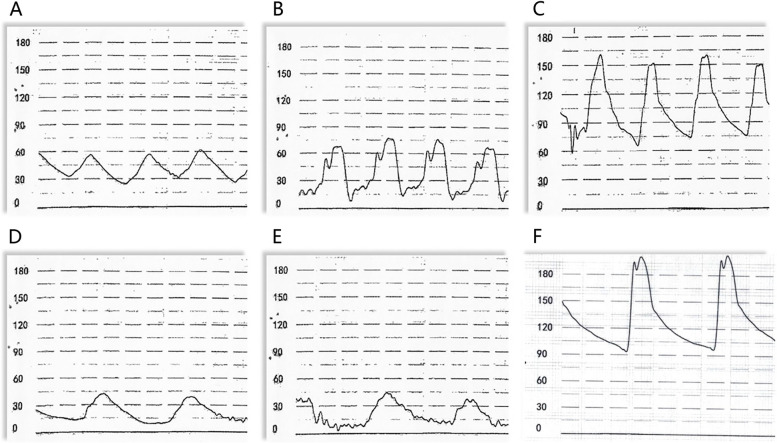
the pressure of cardiac chambers. **A** Pulmonary artery pressure before occlusion; **B** Right ventricular pressure before occlusion; **C** Aortic pressure before occlusion; **D** Pulmonary artery pressure after occlusion; **E** Right ventricular pressure after occlusion; **F** Aortic pressure after occlusion

The patient experienced palpitations and dizziness, with a heart rate of 48 beats per minute and a increased aortic pressure of 200/93 (128) mmHg, Meanwhile, pulmonary artery pressure decreased to 42/10 (20) mmHg (Fig. 1). The ECG displayed sinus bradycardia without ST-segment elevation. After five minutes of observation, the patient's symptoms significantly improved, and the heart rate and blood pressure returned to normal.

After completing the catheterization procedure, we applied compressive dressings to the puncture sites of the right femoral artery and vein to achieve hemostasis. Subsequently, the patient reported experiencing pain in these areas. To evaluate the intensity of the patient's post-procedural discomfort, we utilized the Numerical Rating Scale (NRS). The patient's self-reported pain score was 5, which corresponds to moderate pain at the sites of the femoral artery and vein punctures. Considering this level of pain and based on our clinical judgment, we determined that the administration of analgesic medication was not necessary at that time.

### Twelve hours following the procedure

**Fig. 2:**
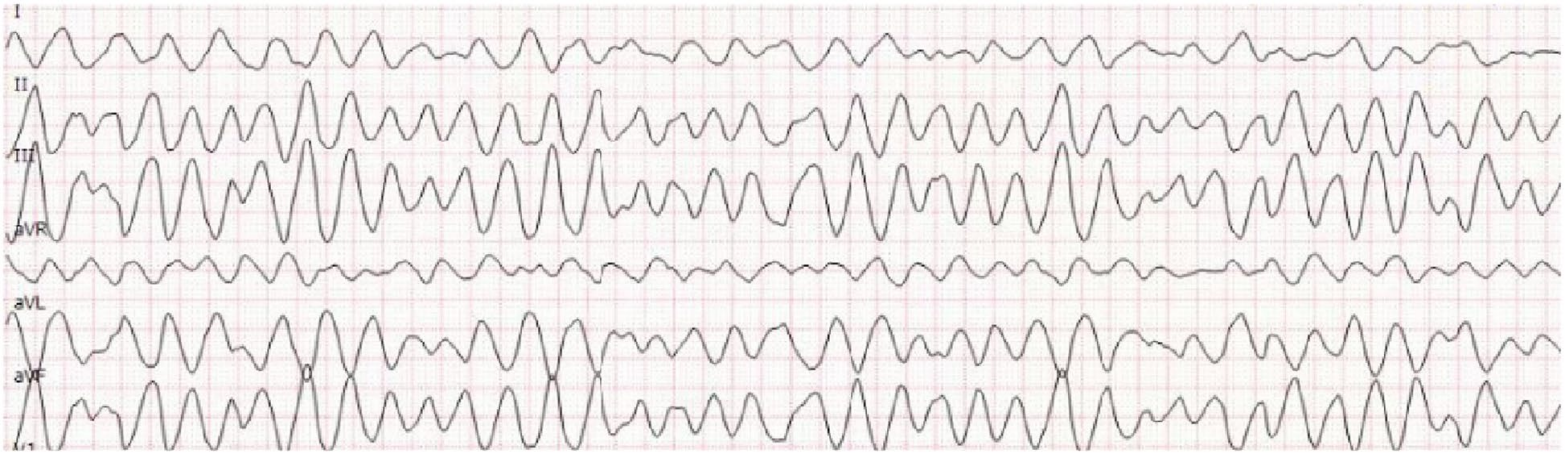
Limb lead electrocardiogram suggestive of ventricular fibrillation

She experienced sudden palpitations and cardiogenic syncope, with ECG monitoring indicating ventricular fibrillation. Immediate electrical defibrillation was performed and simultaneous the limb-lead electrocardiogram still suggested ventricular fibrillation (Fig. 2) and immediate electrical defibrillation again and then ventricular fibrillation ceased, and sinus rhythm was maintained. However, the ECG revealed inverted T waves and a prolonged QTc interval. Troponin levels rose to 20 ng/ml (normal range: 14 ng/ml). NT-ProBNP was 1536 pg/mL (normal range: 300 ng/ml). Subsequently, TTE was conducted, revealing a satisfactory position and shape of the PDA occluder without any residual shunt. However, it indicated left ventricular enlargement and a reduced left ventricular ejection fraction (LVEF) of 31%.

### Two days following the procedure

The ECG revealed T-wave inversion and a further prolongation of the QTc interval, but the patient did not experience any apparent discomfort at this time and declined to undergo coronary angiography and left ventricular angiography.

### Three days following the procedure

**Fig. 3:**
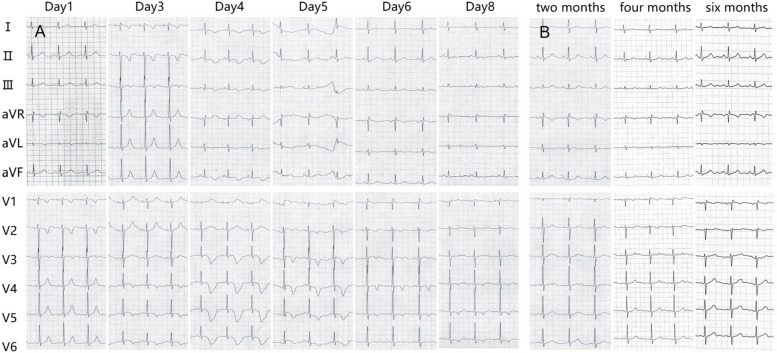
The electrocardiogram change during a 8-day hospitalization (**A**); follow-up ECG changes (**B**)

**Fig. 4:**
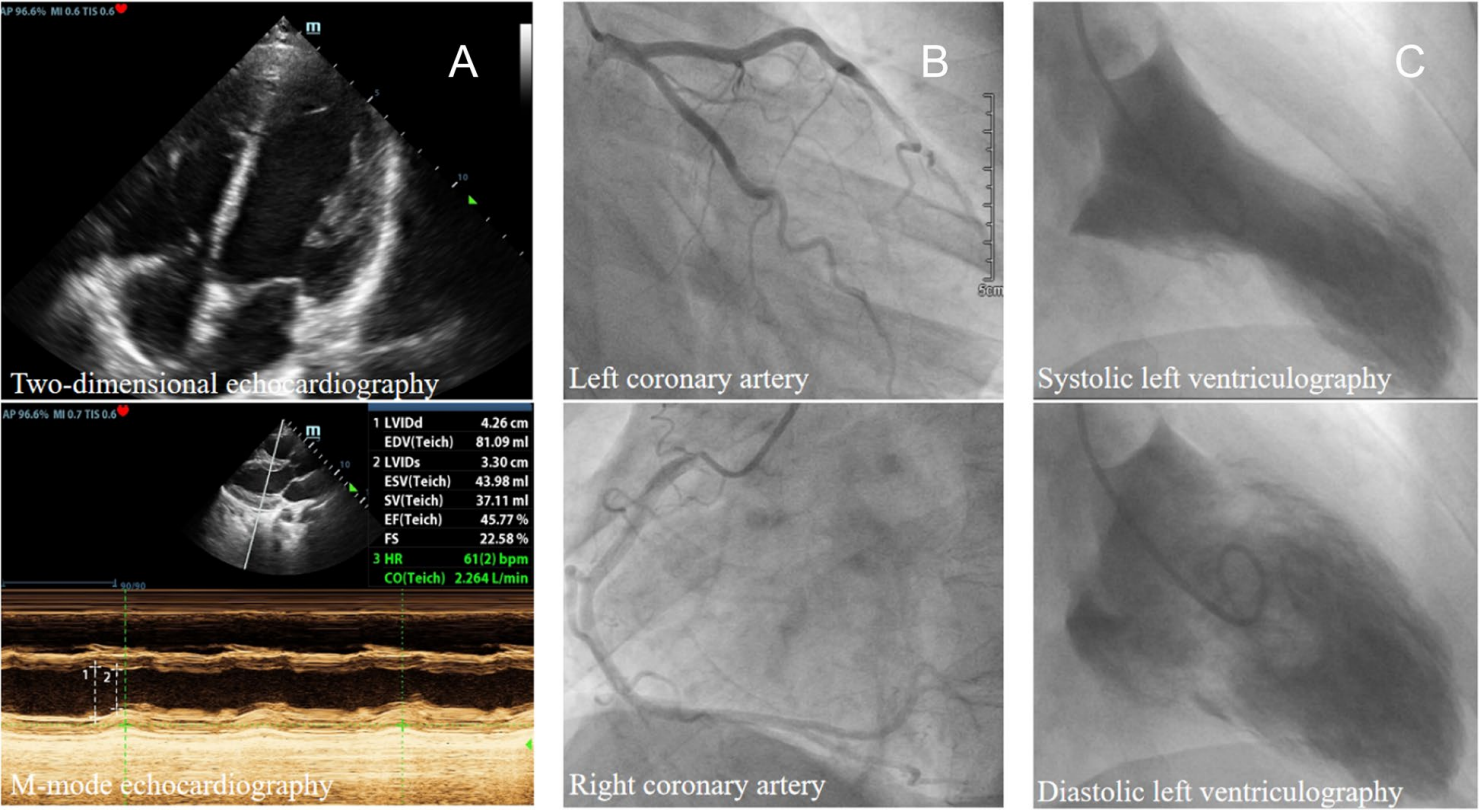
Two-dimensional and M-mode echocardiogram on the third postoperative day (**A**); left and right coronary angiography (**B**); revealed a new left ventricular apex akinesis and apical ballooning during systole (**C**)

The ECG revealed progressive T-wave inversions, and the QTc interval was significantly prolonged to about 700 ms (Fig. 3). TTE showed a decrease in LVEF to 45.8% (Fig. 4A).

On the third day, the patient provided consent for coronary angiography and left ventricular angiography, which revealed no coronary stenosis and TIMI flow grade 3 (Fig. 4B). left ventricular angiography suggests systolic apical balloon-like changes consistent with typical TTS (Fig. 4C). During coronary angiography (performed 48 h after PDA occlusion), it was observed that the coronary arteries exhibited satisfactory blood flow, the occluder maintained its proper shape and position, and no residual shunt was detected. Coronary angiography ruled out concomitant coronary artery disease (CAD) or potential coronary injury during the procedure. The patient received a diagnosis of TTS and was prescribed ACEI and beta blockers.

### Five days following the procedure

The patient received supportive care during the acute phase. ACEI and beta-blocker were maintained post-discharge. Over the course of an eight-day hospital stay, there was a gradual alleviation of symptoms. The patient's condition improved markedly, achieving hemodynamic stability and demonstrating significant improvement in LVEF as well as a resolution of ECG abnormalities (Fig. 3).

### Two months, four months and six months after the procedure

**Fig. 5:**
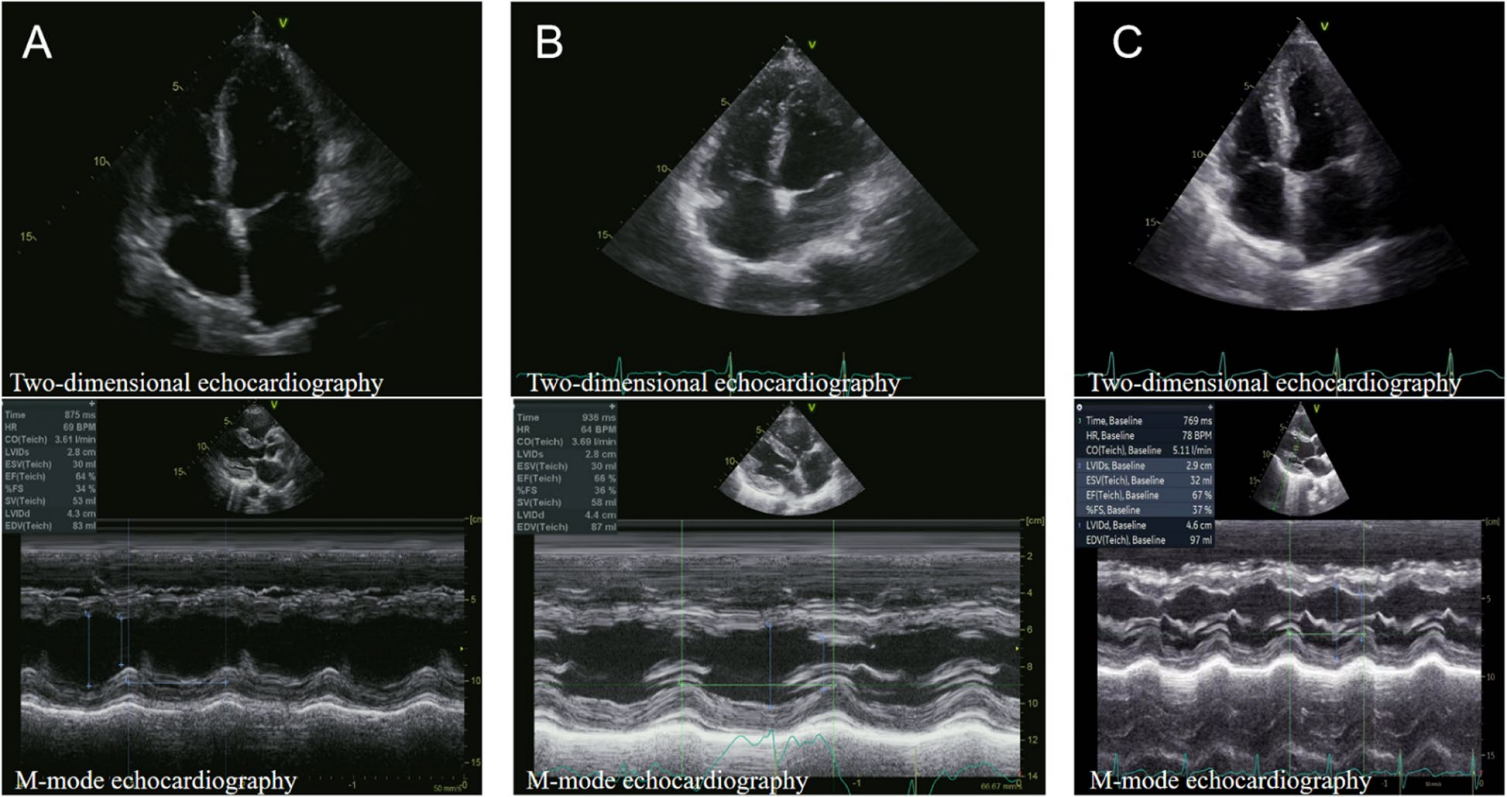
Two-dimensional and M-mode echocardiography at 2-month follow-up (**A**); 4-month follow-up and 6-month follow-up (**C**)

Post-discharge follow-up, the ECG (Fig. 3) and TTE (Fig. 5) demonstrated a gradual return to normal.

## Discussion

TTS is typically characterized by reversible left ventricular dysfunction following emotional or physical stress [4–7]. In this case, the closure of the PDA may have acted as a triggering factor for the development of TTS. Although there have been limited reports of TTS occurring after closure of the PDA [8, 9], to the best of our knowledge, this is a rare case of survival following sudden death after PDA occlusion. The diagnosis of TTS is often challenging due to its broad spectrum of clinical presentations. According to the International Takotsubo Diagnostic Criteria outlined in the 2018 European Heart Journal's International Expert Consensus Document on Takotsubo Syndrome (Part I), the patient's condition met the specified parameters, as detailed in Table 3 [4]. This case basically meets the diagnostic criteria for TTS. The occurrence of TTS in this patient may be related to the interventional therapy employed during the PDA closure.

**Table 3:** International Takotsubo Diagnostic Criteria (InterTAK Diagnostic Criteria

1.Patients show transienta left ventricular dysfunction (hypokinesia, akinesia, or dyskinesia) presenting as apical ballooning or midventricular, basal, or focal wall motion abnormalities. Right ventricular involvement can be present. Besides these regional wall motion patterns, transitions between all types can exist. The regional wall motion abnormality usually extends beyond a single epicardial vascular distribution; however, rare cases can exist where the regional wall motion abnormality is present in the subtended myocardial territory of a single coronary artery (focal TTS)
2. An emotional, physical, or combined trigger can precede the takotsubo syndrome event, but this is not obligatory
3. Neurologic disorders (e.g. subarachnoid haemorrhage, stroke/transient ischaemic attack, or seizures) as well as pheochromocytoma may serve as triggers for takotsubo syndrome
4. New ECG abnormalities are present (ST-segment elevation, ST-segment depression, T-wave inversion, and QTc prolongation); however, rare cases exist without any ECG changes
5. Levels of cardiac biomarkers (troponin and creatine kinase) are moderately elevated in most cases; significant elevation of brain natriuretic peptide is common
6. Significant coronary artery disease is not a contradiction in takotsubo syndrome
7.Patients have no evidence of infectious myocarditis
8. Postmenopausal women are predominantly affected

The stimulation of the sympathetic nervous system plays a significant role in the pathogenesis of TTS [10, 11]. However, in cases where there is no previous history of sympathetic nervous system stimulation, the underlying mechanism may be hindered by an increase in cardiac vagus nerve tension [12, 13]. In the context of the closure of the PDA, it is possible that the suppression of PDA could lead to stimulation of both the sympathetic and parasympathetic nervous systems, as they are distributed around the PDA [7, 14]. Following successful closure, the patient experienced a transient decrease in heart rate and an increase in descending aortic blood pressure, which further supports this hypothesis.

Certainly, another potential factor contributing to TTS in this case is the sudden decrease in pulmonary artery pressure [7]. Changes in arterial blood pressure lead to disturbances in the autonomic nervous system and promote the development of catecholamine storms [15, 16]. We propose a hypothesis that the tension of sympathetic and parasympathetic nerves and micro vascular dysfunction could undergo changes in response to pressure variations within the heart and the major arteries, ultimately leading to TTS. It is imperative to emphasize that, although we have hypothesized pain as a possible trigger for TTS in this instance, this association remains speculative. The results of our study provide a basis for additional investigation, which is crucial in determining whether pain can be conclusively identified as a precipitating factor for TTS.

## Conclusion

We present a case of TTS following the closure of the PDA. The occurrence of TTS may indeed be associated with the intervention performed. Moving forward, we aim to continue observing and investigating the underlying mechanisms involved in TTS during the interventional treatment of CHD.

### Supplementary Information

**Additional file 1:** **Video 1:** This video demonstrates the aortic angiography results, indicating the presence of PDA with left-to-right shunting. **Video 2:** This video illustrates the occlusion process of the PDA, showing the successful deployment of the occlusion device resulting in the occlusion of the ductus arteriosus. **Video 3:** After completion of the occlusion procedure, the occlusion device maintains an optimal morphology.

## Data Availability

All relevant data supporting the conclusions of this article are included within the article.

## Abbreviations

ECG: Electrocardiogram
TTS: Takotsubo syndrome
CHD: Congenital heart disease
PDA: Patent ductus arteriosus
TTE: Transthoracic echocardiography
LVEF: Left ventricular ejection fraction
CAD: Coronary artery disease
ACEI: Angiotensin converting enzyme inhibitor
