# Supplementary material for: Is Takotsubo syndrome induced by patent ductus arteriosus occlusion?
Source: BMC Cardiovasc Disord. 2024 Mar 2;24:135. doi: 10.1186/s12872-024-03788-0 (PMC10908159; doi:10.1186/s12872-024-03788-0)
Supplement: Supplementary file 1 — Additional file 1: Video 1: This video demonstrates the aortic angiography results, indicating the presence of PDA with left-to-right shunting. Video 2: This video illustrates the occlusion process of the PDA, showing the successful deployment of the occlusion device resulting in the occlusion of the ductus arteriosus. Video 3: After completion of the occlusion procedure, the occlusion device maintains an optimal morphology. [file 12872_2024_3788_MOESM1_ESM.pptx]

## Slide 1
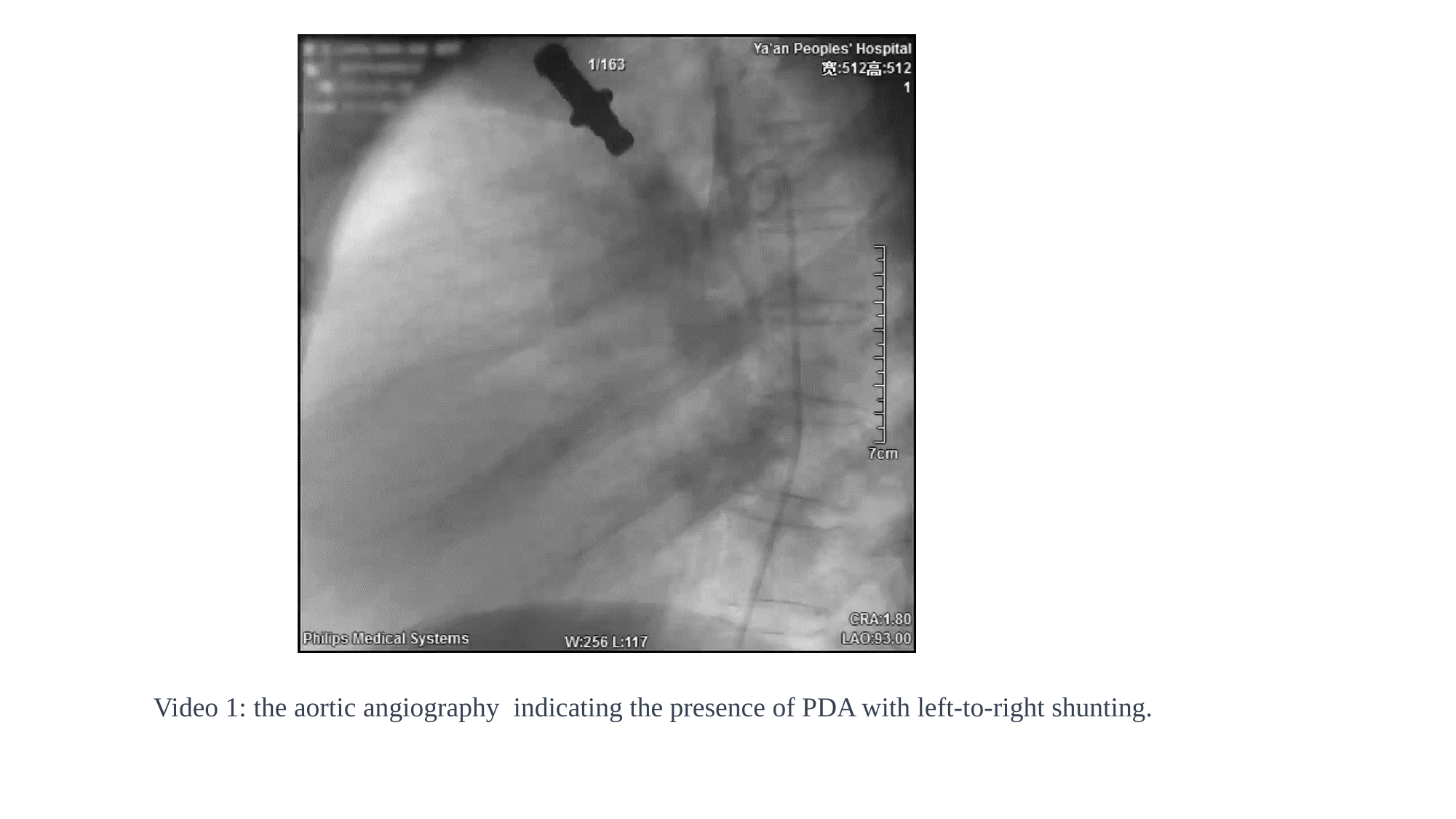

Video 1: the aortic angiography indicating the presence of PDA with left-to-right shunting.

## Slide 2
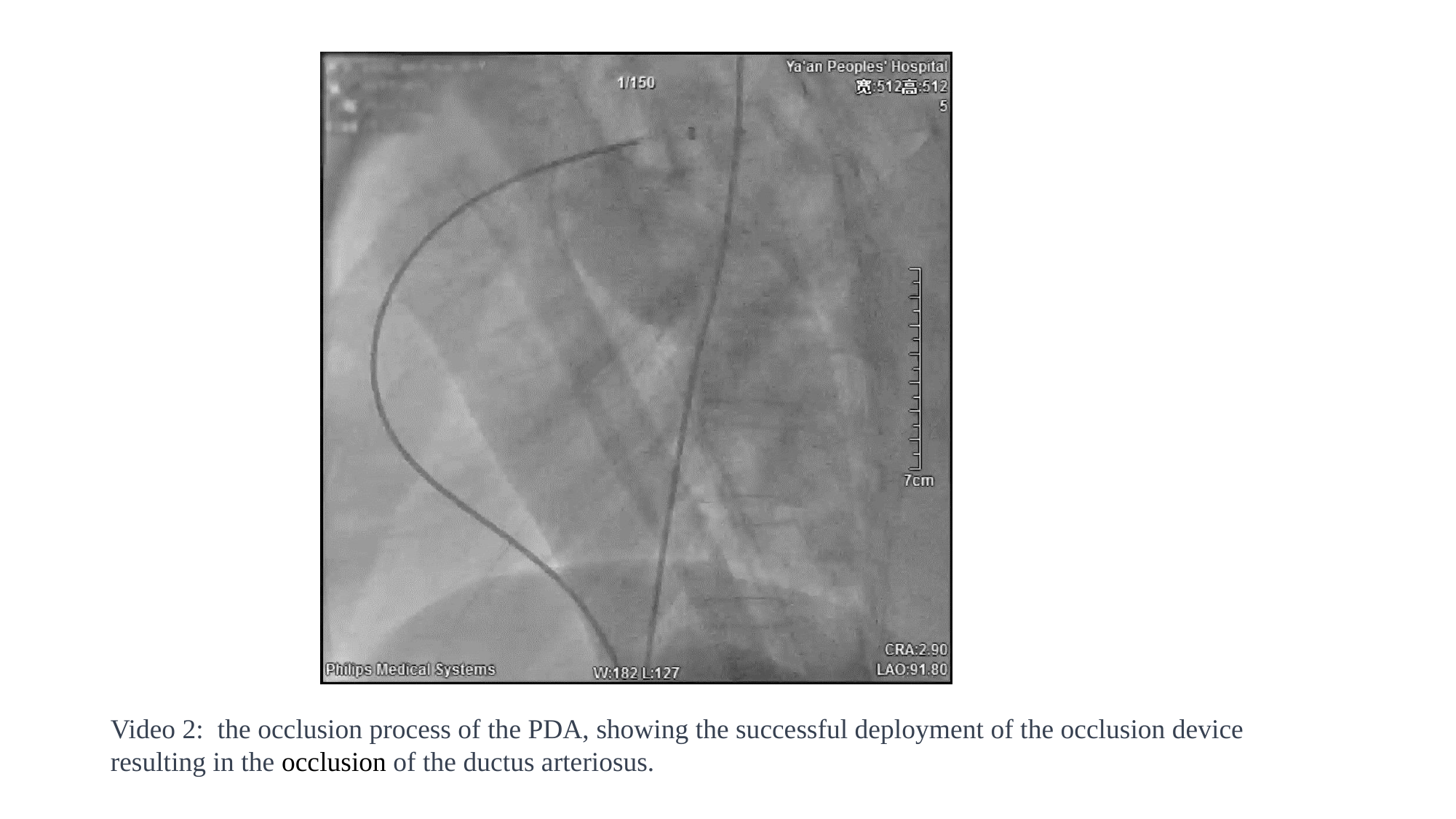

Video 2: the occlusion process of the PDA, showing the successful deployment of the occlusion device resulting in the occlusion of the ductus arteriosus.

## Slide 3
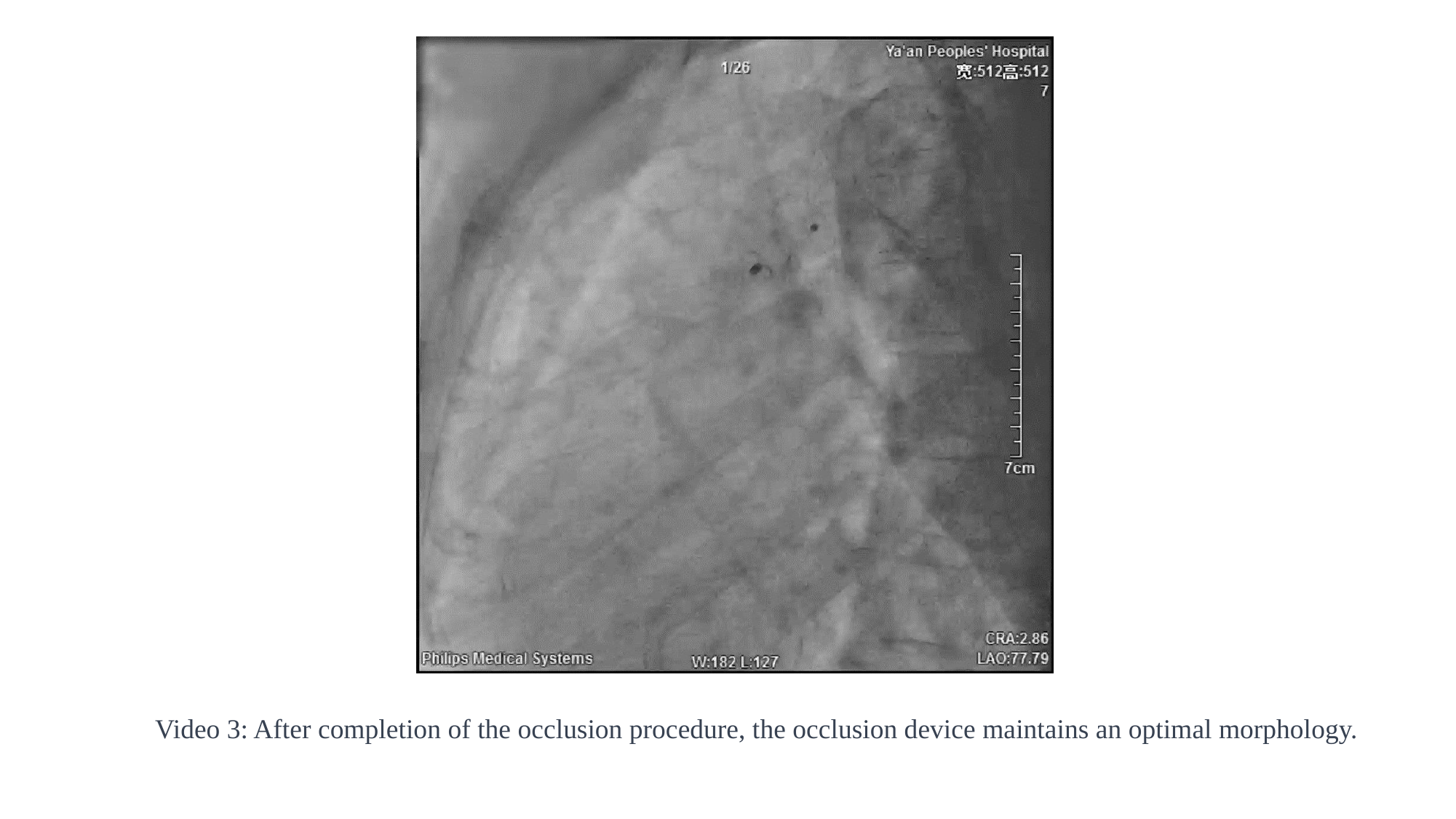

Video 3: After completion of the occlusion procedure, the occlusion device maintains an optimal morphology.
